# Survival analysis of patients with subglottic squamous cell carcinoma based on the SEER database

**DOI:** 10.1016/j.bjorl.2021.09.001

**Published:** 2021-10-19

**Authors:** Fan Yang, Lu He, Yuansheng Rao, Yanjun Feng, Jianhong Wang

**Affiliations:** a Department of Otorhinolaryngology Head and Neck Surgery, Beijing Anzhen Hospital, Capital Medical University, Beijing, China; b Department of Otorhinolaryngology Head and Neck Surgery, Beijing Friendship Hospital, Capital Medical University, Beijing, China

**Keywords:** Subglottic laryngeal squamous cell carcinoma, Surveillance, epidemiology and end results (SEER), Survival, Treatment, Surgery, Adjuvant therapy

## Abstract

**Objective:**

This study aimed to investigate the demographic and clinicopathological characteristics, and survival outcomes of subglottic Squamous Cell Carcinoma (SCC) based on the Surveillance, Epidemiology, and End Results (SEER) database.

**Methods:**

Demographic and clinicopathological information, including age, sex, race, tumor size, histologic grade, clinical/TNM stage, tumor invasion extent, Lymph Node Metastasis (LNM) extent, size of metastatic lymph nodes, LNM ratio and treatment data, of 842 subglottic SCC patients diagnosed between 1996 and 2016 were acquired. Kaplan-Meier survival analyses were performed to assess the effects of clinicopathological characteristics, treatment modalities, surgical procedures, and adjuvant therapies on overall survival and cancer-specific survival.

**Results:**

Subglottic SCC was more frequent among males aged 60–70 years, with low-grade but locally advanced lesions without local or distant metastases. Age and several primary tumor/LNM related variables were independent risk factors for overall survival and cancer specific survival. Advanced-stage and high-grade disease led to unfavorable prognosis. The most common treatment modality and surgical procedure were surgery plus radiotherapy and total laryngectomy, respectively. Surgery plus radiotherapy provided favorable 5-year survival outcomes, while total laryngectomy had the worst. Surgery plus adjuvant therapy showed better survival outcomes than surgery alone.

**Conclusion:**

This study confirmed the rarity of subglottic SCC. Patients with subglottic SCCs suffered poor prognosis especially for those with advanced-stage or high-grade lesions. The prognosis of subglottic SCC remained poor over the years, despite recent progress in cancer therapies. Surgery plus adjuvant therapy improved the survival outcome. Although larynx preservation surgery was beneficial for early-stage disease, total laryngectomy was favored for patients with advanced tumors.

**Level of evidence:**

Level 4.

## Highlights

• Prognosis of subglottic squamous cell carcinoma remained poor over the years.
• Combination of surgery and adjuvant therapies improved the survival outcomes.
• Early-stage tumors benefited from larynx preservation surgery; advanced tumors benefited from total laryngectomy.

## Introduction

Primary subglottic Squamous Cell Carcinoma (SCC) is a rare malignancy accounting for only 1%–3% of all laryngeal carcinomas.^1^, ^2^, ^3^ It is generally asymptomatic at earlier stages and typically presents at advanced stages, leading to substantially worse prognosis than Laryngeal Squamous Cell Carcinomas (LSCCs) originated from supraglottic and glottic.^3^, ^4^ Despite recent progress in treatment of laryngeal carcinoma, no consensus on the optimal subglottic SCC management has been reached.^5^ The therapeutic strategy varies depending on the stage of disease, which usually containing surgery, adjuvant therapy, or a combination of both. Mounting evidence suggests that early-stage subglottic SCCs can be managed with a single type of treatment, whereas advanced-stage tumors require combined-modality therapy.^6^, ^7^, ^8^ Although radiotherapy preserves laryngeal function, its ability to improve long-term patient survival remains controversial.^9^, ^10^, ^11^ Chemotherapies, including induction Chemotherapy and Concurrent Chemoradiotherapy (CCRT), are widely used to treat laryngeal carcinoma; however, their clinical usefulness in patients with subglottic SCC remains unclear.^12^, ^13^, ^14^ In this study, we conducted a retrospective analysis using data from the National Cancer Institute’s Surveillance, Epidemiology, and End Results (SEER) database to investigate the demographic and clinicopathological characteristics as well as survival outcomes of subglottic SCC.

## Methods

### Data source and study subjects

We collected data from the SEER database pertaining to cases from 1996 to 2016 in the United States. According to the International Classification of Diseases for Oncology, data with a confirmed histological diagnosis of laryngeal SCC were extracted using the SEER code (histology recode) 8050–8089 (squamous cell neoplasms). Patients with subglottic lesions (anatomic site code C32.3) were then selected.

Demographic data, including sex, age at diagnosis, year of diagnosis, and ethnicity, were collected. The years of diagnosis were grouped into 1996–2006 and 2007–2016. The clinicopathological variables, including tumor size, histologic grade, tumor invasion extent, Lymph Node Metastasis (LNM) extent, average size of metastatic lymph nodes, LNM ratio (number of metastatic lymph node excised/total number of lymph nodes excised), and TNM/clinical stage per the American Joint Committee on Cancer staging system, were collected, and analyzed. Treatment modality, surgical procedure, survival time, Overall Survival (OS) status, and Cancer-Specific Survival (CSS) status were also collected. All variables were defined using the SEER specific codes. The histologic grade was redefined as low-grade (well and moderately differentiated) or high-grade (poorly differentiated and undifferentiated). The clinical stage was redefined as early-stage (stages I and II) or advanced-stage (stages III and IV).

### Statistical methods

Descriptive statistics were calculated means and standard deviations (n ± SD), categorical data were expressed as percentages. Patient survival was estimated using the Kaplan-Meier method, and comparisons between groups were made using the log-rank test. The multivariate Cox proportional hazards model was used to analyze the relationship between demographic plus clinicopathological variables, and OS and CSS. The Hazard Ratio (HR) and Confidence Intervals (CI) were calculated. Two-tailed *p*-values less than 0.05 were considered statistically significant. SEER data were extracted using the SEER*Stat 8.3.6 (National Cancer Institute, Bethesda, MD, USA). Statistical analyses were performed using SPSS 25.0 (IBM Corp., Armonk, NY, USA) and R (v3.5.1) with packages survival, survminer, ggplot2.

### Ethical statement

The study was approved by the ethical review board of the institution (NO. 2021100X) and complied with the ethical standards of the Declaration of Helsinki, as well as with relevant national and international guidelines.

## Results

### Demographic and clinicopathological characteristics

This cohort included 842 patients with subglottic SCC acquired from the SEER database. The mean age at diagnosis was 65.88 ± 11.58 years. Most patients were aged between 60 and 69 years (n = 267, 31.7%). Male gender was more common with a 3.98:1 ratio as compared to female gender. White patients accounted for 77.4%; 19.0% and 3.6% of patients were black individuals or individuals of other races. The average tumor size was 28.76 ± 15.12 mm and the average metastatic lymph node size was 25.5 ± 13.68 mm. Of the 260 cases with available total and positive metastatic number of lymph node excised, the LNM ratio was 12.03%. 513 (60.9%) cases were low-grade, with 186 (22.1%) with high-grade, and 143 (17.0%) without detailed histological data. Of the 573 patients with available clinical stage information, 194 (23.0%) had early-stage disease, and 379 (45.0%) had advanced-stage disease. The majority of the patients presented with T4 disease (n = 256, 30.4%), without cervical lymphatic involvement (N0, n = 422, 50.1%) and distant metastasis (M0, n = 549, 65.2%). For the overall treatment type, 413 (49.0%) received surgical treatment, 337 (40.0%) received non-surgical treatment and 92 (10.9%) were untreated. 478 (56.8%) patients were diagnosed between 1996 and 2006, and 364 (43.2%) between 2007 and 2016. The average survival time was 45.55 ± 49.55 months (Table 1**)**.

**Table 1:** Demographic and clinicopathological characteristics of patients with subglottic squamous cell carcinoma.

Variables	n (%)
Overall	842 (100%)
Survival months (mean ± SD)	45.55 ± 49.55
Tumor size (mean ± SD, mm)	28.76 ± 15.12
Size of metastatic lymph node (mean ± SD, mm)	25.5 ± 13.68
Age at diagnosis (mean ± SD, yrs)	65.88 ± 11.58
Age groups	
0–19	0 (0%)
20–29	2 (0.2%)
30–39	6 (0.7%)
40–49	44 (5.2%)
50–59	203 (24.1%)
60–69	267 (31.7%)
70–79	206 (24.5%)
80–89	99 (11.8%)
90+	16 (1.9%)
Sex	
Female	169 (20.1%)
Male	673 (79.9%)
Race	
White	652 (77.4%)
Black	160 (19.0%)
Other	30 (3.6%)
Histologic grade	
Low-grade	513 (60.9%)
Well differentiated	75 (8.9%)
Moderately differentiated	438 (52.0%)
High-grade	186 (22.1%)
Poorly differentiated	182 (21.6%)
Undifferentiated	4 (0.5%)
Unknown	143 (17.0%)
Clinical stage	
Early-stage	194 (23.0%)
Stage I	86 (10.2%)
Stage II	108 (12.8%)
Advanced stage	379 (45.0%)
Stage III	85 (10.1%)
Stage IV	294 (34.9%)
Unknown	269 (31.9%)
T stage	
T1	105 (12.5%)
T2	127 (15.1%)
T3	91 (10.8%)
T4	256 (30.4%)
Tx	263 (31.2%)
N stage	
N0	422 (50.1%)
N1	63 (7.5%)
N2	80 (9.5%)
N3	9 (1.1%)
Nx	268 (31.8%)
M stage	
M0	549 (65.2%)
M1	34 (4.0%)
Mx	259 (30.8%)
Overall treatment	
Surgical treatment	413 (49.0%)
Non-surgery treatment	337 (40.0%)
None	92 (10.9%)
Years of diagnosis	
2007–2016 (10 years)	478 (56.8%)
1996–2006 (11 years)	364 (43.2%)

### Treatment data

Treatment modalities were characterized as Surgery (S) alone, Radiotherapy (RT) alone; Chemotherapy (CT) alone; combined surgery, RT, and CT (S + RT + CT); combined surgery and RT (S + RT); combined surgery and CT (S + CT); combined RT and CT (RT + CT); and no treatment. Among the 750 patients with detailed information, the majority of whom received S + RT (n = 193, 25.7%), followed by RT + CT (n = 159, 21.2%), RT alone (n = 151, 20.1%) and surgery alone (n = 118, 14.0%). Only 27 (3.6%) patients received CT alone and 4 (0.5%) received S + CT. Of the 413 patients who underwent surgery, 131 underwent local tumor excision, 22 underwent partial laryngectomy, 227 underwent total laryngectomy, while surgery information of 33 patients were unspecified. A total of 97 patients who underwent local tumor excision also received adjuvant therapy (radiotherapy, chemotherapy or both), compared with 14 who underwent partial laryngectomy, and 156 who underwent total laryngectomy.

### Analysis of risk factors for OS and CSS

Univariate analysis revealed that advanced age, extensive tumor invasion, extensive LNM, higher LNM ratio and larger tumor size associated significantly with poor OS. Male sex, different race, high-grade tumor, larger metastatic lymph node size were not risk factors for OS. Multivariate analysis showed that all of these significant factors independently predicted poor OS (Table 2).

**Table 2:** Univariate and multivariate analysis of risk factors associated with OS and CSS.

Variable	OS				CSS			
	Univariate^a^		Multivariate^a^		Univariate^a^		Multivariate^a^	
	HR (95%CI)	*p*-value	HR (95%CI)	*p*-value	HR (95%CI)	*p*-value	HR (95%CI)	*p*-value
Age	1.036 (1.028–1.044)	< 0.001	1.027 (1.015–1.039)	< 0.001	1.009 (0.997–1.020)	0.142		
Sex	1.073 (0.872–1.320)	0.507			1.433 (1.017–2.019)	< 0.05	1.390 (0.959–2.015)	0.082
Race	0.874 (0.728–1.049)	0.148			0.834 (0.633–1.097)	0.195		
High-grade tumor	1.207 (0.989–1.473)	0.064			1.607 (1.216–2.124)	< 0.01	1.673 (1.255–2.230)	< 0.001
Extensive tumor invasion	1.001 (1.000–1.002)	< 0.001	1.002 (1.001–1.003)	< 0.001	1.003 (1.001–1.005)	< 0.001	1.001 (1.000–1.002)	< 0.001
Tumor size	1.014 (1.005–1.023)	< 0.01	1.016 (1.007–1.026)	< 0.01	1.035 (1.021–1.048)	< 0.001	1.026 (1.011–1.042)	< 0.01
Extensive LNM	1.002 (1.001–1.003)	< 0.001	1.003 (1.002–1.003)	< 0.001	1.005 (1.002–1.008)	< 0.001	1.004 (1.002–1.006)	< 0.01
LNM ratio	2.840 (1.469–5.489)	<0.01	2.909 (1.507–5.612)	<0.01	3.664 (1.531–8.768)	<0.01	4.251 (1.709–10.575)	<0.01
Metastatic lymph node size	1.002 (0.987–1.018)	0.766			1.012 (0.993–1.032)	0.221		

HR, Hazard Ratio; 95% CI, Confidence Interval.

a Cox proportional hazards model.

As for CSS, univariate analysis revealed male sex, high-grade tumor, extensive tumor invasion, extensive LNM, higher LNM ratio and larger tumor size associated significantly with poor CSS. Advanced age, different race and larger metastatic lymph node size were not risk factors for CSS. Multivariate analysis showed that all of these significant factors independently predicted poor CSS, except male sex (Table 2).

### Survival analysis

**Figure 1:**
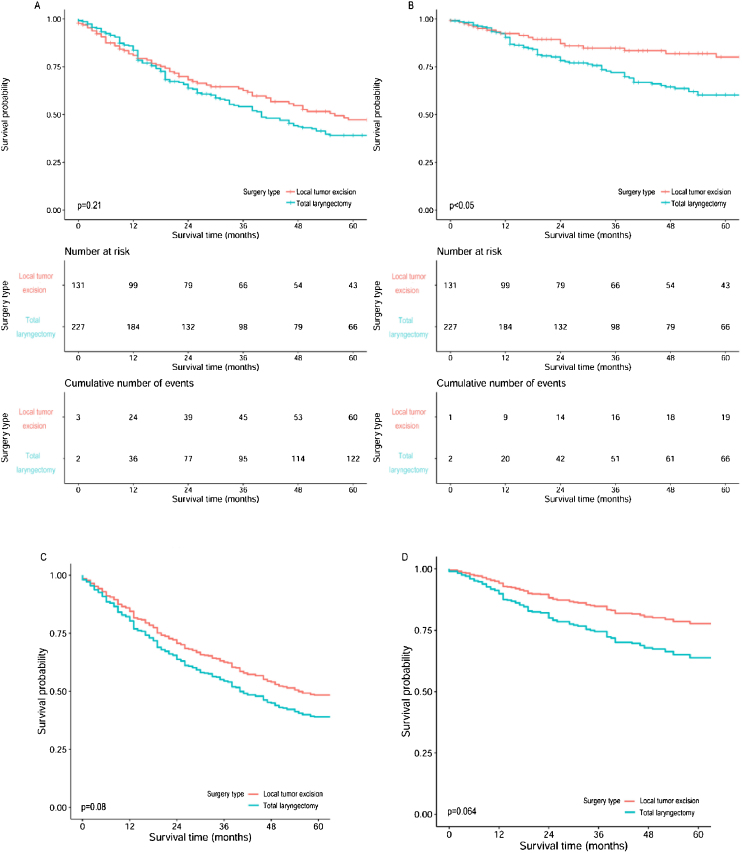
Survival curves of OS and CSS for subglottic SCC compared between local tumor excision and total laryngectomy. (A) Overall survival curves, (B) Cancer specific survival curves, (C) Overall survival curves after adjusted for age and T staging, (D) Cancer specific survival curves after adjusted for age and T staging.

The overall 5-year OS and CSS rates of the 842 patients were 39.4% and 64.6%, respectively. Survival analysis after patient stratified by treatment modalities revealed that S + RT led to the best 5-year OS (51.3%) and CSS (71.6%); on the contrary, CT alone had the worst 5-year OS (18.5%) and CSS (42.6%). With regard to surgical procedures, partial laryngectomy led to the highest 5-year OS (47.6%) rate, and total laryngectomy led to the worst (39.1%); moreover, local tumor excision led to the highest 5-year CSS (80.1%) rate, and again, total laryngectomy led to the worst (60.3%). Further comparison of the survival between local tumor excision and total laryngectomy, after adjusted for age and T stage, also demonstrated much better outcomes for local tumor excision (Fig. 1). Furthermore, surgery plus adjuvant therapy led to higher 5-year OS and CSS than surgery alone in local tumor excision subgroup and total laryngectomy subgroup; and the opposite results were observed in partial laryngectomy subgroup (Table 3).

**Table 3:** The survival of subglottic SCC based on treatment modality, surgical procedure, and surgery/surgery plus adjuvant therapy.

Variables	5-year OS				5-year CSS			
	Overall (%)	1996–2006 (%)	2007–2016 (%)	*p*-value^b^	Overall (%)	1996–2006 (%)	2007–2016 (%)	*p*-value^b^
Overall	39.4	36.7	42.1	0.436	64.6	60.0	68.8	0.187
Treatment modality								
S	32.3	28.8	38.0	0.595	64.6	59.2	72.5	0.322
S + RT + CT	39.8	37.5	41.2	0.294	60.6	54.1	62.5	0.123
S + RT	51.3	47.7	56.3	0.365	71.6	65.9	80.3	0.105
S + CT	50.0	n/a	50.0	n/a	50.0	n/a	50.0	n/a
RT	42.8	43.3	42.9	0.632	72.7	74.9	71.1	0.429
CT	18.5	11.1	22.2	0.670	42.6	37.0	47.8	0.294
RT + CT	43.9	31.4	50.6	0.052	59.0	43.4	67.3	0.063
*p*-value^a^	< 0.001	< 0.05	< 0.01		< 0.01	< 0.001	< 0.05	
Surgical procedure								
Local tumor excision	47.3	39.0	56.0	0.066	80.1	77.1	83.1	0.971
Partial laryngectomy	47.6	53.3	33.3	0.154	63.5	64.0	66.7	0.422
Total laryngectomy	39.1	38.7	39.3	0.940	60.3	56.0	64.4	0.097
* p*-value^a^	0.417	0.893	< 0.05		< 0.05	0.062	<0.05	
S vs. S + AT^e^								
Local tumor excision								
S	28.5	13.3	45.3	0.081	75.0	69.2	75.0	0.635
S + AT	53.7	47.7	59.5	0.236	82.0	78.0	85.8	0.782
* p*-value^a^	< 0.01	< 0.01	0.169		0.387	0.958	0.274	
Partial laryngectomy								
S	50.0	57.1	0.00	0.128	80.0	80.0	100	0.323
S + AT	46.2	50.0	40.0	0.396	52.7	50.0	60.0	0.688
* p*-value^a^	0.575	0.727	0.897		0.400	0.671	0.502	
Total laryngectomy								
S	31.8	31.4	33.5	0.795	58.5	52.9	67.6	0.317
S + AT	42.4	42.1	41.8	0.945	61.1	57.2	63.4	0.168
* p*-value^a^	0.225	0.743	0.132		0.932	0.856	0.935	

S, Surgery; RT, Radiotherapy; CT, Chemotherapy; S + AT, Surgery plus Adjuvant Therapy.

a Log-rank test based on treatment modality, surgical procedure, and surgery/surgery plus adjuvant therapy.

b Log-rank test based on decades of diagnosis.

Between the two years-of-diagnosis groups, the differences of patients’ overall 5-year OS and CSS rates were non-significant; and similar results were observed when stratified by treatment modalities, surgical procedures, and surgery versus surgery plus adjuvant therapies. Statistically significant differences were found for the 5-year survival among patients receiving different treatment modalities within each years-of-diagnosis subgroup; while significant differences of 5-year survival among those treated with different surgeries were only observed between 2007 and 2016. Survival results of patients treated with surgery alone differed non-significantly from those who also received adjuvant therapy in each time subgroup, where exception was found for 5-year OS between 1996 and 2006 in the local tumor excision subgroup (Table 3).

**Figure 2:**
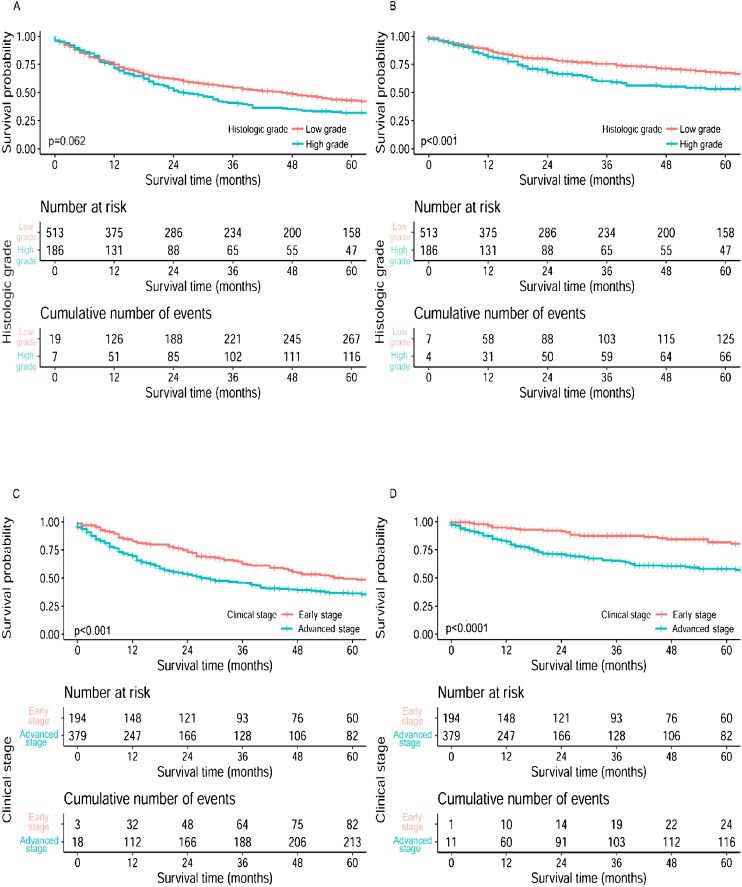
Survival curves of OS and CSS for subglottic SCC stratified by histologic grade and clinical stage. (A) Overall survival curves by histologic grade, (B) Cancer specific survival curves by histologic grade, (C) Overall survival curves by clinical stage, (D) Cancer specific survival curves by clinical stage.

Patients with low-grade or early-stage disease showed statistically better CSS than those with high-grade or advanced-stage disease. Statistically difference of OS was only found in the comparison between early and advanced stage disease (Fig. 2). Regardless of the treatment types, patients with low-grade tumors still showed much better higher 5-year survival, except for those treated with CT alone; patients at early-stage showed much better 5-year survival than advanced-stage patients for all treatment types. Survival analyses based on surgical procedures revealed low-grade tumors had better survival results than high-grade tumors in the local tumor excision subgroup and total laryngectomy subgroup, where the opposite result was revealed in partial laryngectomy subgroup. Patients at early-stage also had higher 5-year survival than those at advanced-stage in all three surgical procedure subgroups. As for the comparison between surgery alone and surgery plus adjuvant therapy, most patients with low-grade or early-stage disease showed better survival outcomes than those with high-grade or advanced-stage disease respectively, no matter if they received adjuvant therapy (Table 4).

**Table 4:** Effect of clinical stage and histologic grade on the survival of subglottic SCC.

Variables	5-year OS^a^						5-year CSS^a^					
	Early - stage (%)	Advanced - stage (%)	*p*-value^b^	Low - grade (%)	High - grade (%)	*p*-value^b^	Early - stage (%)	Advanced - stage (%)	*p*-value^b^	Low - grade (%)	High - grade (%)	*p*-value^b^
Overall	49.3	36.4	< 0.001	42.9	31.9	0.062	81.8	58.0	< 0.001	67.4	53.2	<0.01
Treatment modality												
S	48.1	28.7	0.075	33.2	31.9	0.966	100	58.4	< 0.01	63.7	60.4	0.970
S + RT + CT	64.8	39.4	0.198	45.1	32.3	< 0.05	74.1	60.7	0.591	71.0	45.4	<0.05
S + RT	59.9	53.4	0.606	58.5	34.6	< 0.05	93.4	66.7	< 0.05	77.6	48.9	<0.01
S + CT	n/a	50.0	n/a	100	0.00	0.317	n/a	50.0	n/a	100	0.00	0.317
RT	44.4	27.3	< 0.01	46.0	20.7	0.172	79.2	55.6	<0.01	69.1	62.7	0.993
CT	25.0	21.4	0.753	14.3	50.0	0.128	66.7	45.6	0.749	36.0	75.0	0.637
RT + CT	56.7	43.4	0.298	49.2	43.3	0.362	66.1	60.4	0.708	64.7	59.1	0.237
Surgical procedure												
Local tumor excision	57.5	43.2	0.152	48.6	38.5	0.332	91.3	58.4	<0.01	83.8	55.3	<0.01
Partial laryngectomy	50.0	14.3	0.179	43.8	66.7	0.195	50.0	42.9	0.460	58.3	66.7	0.373
Total laryngectomy	41.8	40.0	0.780	46.0	28.1	<0.05	87.5	64.7	0.299	67.5	45.0	<0.01
S vs. S + AT												
Local tumor excision												
S	50.0	14.6	< 0.05	25.7	22.2	0.955	100	24.3	< 0.01	66.5	55.6	0.440
S + AT	59.8	51.5	0.533	55.6	43.8	0.246	89.3	66.0	< 0.05	87.7	56.1	<0.01
Partial laryngectomy												
S	100	0.00	0.317	57.1	n/a	n/a	100	100	n/a	80.0	n/a	n/a
S + AT	100	16.7	0.637	33.3	66.7	0.144	100	33.3	0.809	41.7	66.7	0.391
Total laryngectomy												
S	30.0	31.1	0.535	34.4	31.9	0.663	100	62.3	0.211	65.2	55.4	0.611
S + AT	50.0	46.1	0.633	53.2	25.9	<0.01	75.0	65.7	0.743	73.1	39.1	<0.01

S, Surgery; RT, Radiotherapy; CT, Chemotherapy; S + AT, Surgery plus adjuvant therapy.

a Only cases with available stage and histologic grade data.

b Log-rank test.

## Discussion

Subglottic SCC is a rare entity accounting for less than 5% of all LSCC.^15^, ^16^, ^17^ We acquired data of LSCC between 1996 and 2016 since meticulous records were just starting to be collected into the SEER database in recent decades, not to mention the rapidly development of management towards laryngeal carcinoma. We confirmed the rarity of subglottic SCC: a total of 48,521 laryngeal SCC cases were registered, with only 842 (1.7%) subglottic SCC cases. Most studies on subglottic SCC involved small cohorts for its rarity, making it difficult to establish the incidence and the underlying mechanisms.^18^ The imprecise definition of the superior subglottic border, accidental sampling of subglottic-invading glottic SCC, and undetermined origins of advanced-stage disease further challenge the elucidation of the clinicopathological characteristics of subglottic SCC.^19^, ^20^

Numerous studies have shown that men are more susceptible to LSCC than women due to the much commonly exposures to environmental risk factors, for instance tobacco.^21^, ^22^ Consistently, we found subglottic SCC is more frequent among white males aged 60–70 years, and those at advanced age suffered unfavorable overall survival outcomes.^4^, ^7^, ^16^ The average tumor size was 28.76 mm in current study. Considering these data may have been derived from surgical specimens, they might not to be representative of actual tumor size; but we still verified that larger tumor is independent risk factor for both OS and CSS, which reinforced the notoriously consensus that local staging of LSCC is proportional to tumor size.^23^ Although high-grade lesion was found in only 22.1% cases, it did have a devastating effect on CSS. Admittedly, pathological staging represents important information and should be included in staging, it doesn’t supplant clinical staging as the primary scheme yet.^24^ Among patients with available clinical information, most of whom were at advanced stage which were primarily due to T4 disease, reflected by 18.1% patients presented cervical lymphatic involvement. Our results agreed with the prevailing studies as despite the locally advanced invasion at presentation, subglottic SCCs may infrequently present with cervical lymphatic involvement with reported incidences ranged from 4% to 21.5%.^3^, ^25^, ^26^, ^27^ Subglottic SCCs prone to escape through the intercartilagenous infrastructure rather than invading across the cartilaginous boundaries of larynx, result in disease progression without overt early symptoms and missed diagnosis during imaging examination.^4^ Subsequent analysis of risk factors confirmed the independently predictive effects of tumor invasion extent and LNM extent, which directly related to the staging and prognosis of LSCC.^5^, ^16^ The lymphatic drainage pathway of the subglottis starts from prelaryngeal (Delphian) or pretracheal lymph nodes to paralaryngeal or paratracheal then crosses the midline, allowing for metastases through-out level VI to the lateral neck, and higher prevalence of LNM could lead to impaired survival.^20^, ^28^ Our finding validated these studies as the higher the positive ratio of metastatic lymph nodes, the worse the OS and CSS rates. However due to incomprehensive distribution of data, deeply exploration of survival outcomes with certain-area invasion, for example cricoid cartilage and prelaryngeal/paralaryngeal lymph nodes where subglottic LSCCs were reported easier to infringe, was unable to carry out.^29^, ^30^ Unfortunately, this research attempted but failed to investigate if the neurovascular invasion or extranodal extension had associations with poor prognosis in subglottic SCC because of data missing in the SEER database.

The aggressive behaviors of subglottic SCC are featured as predilection for laryngotracheal cartilage, extra-laryngeal invasion, high incidence of paratracheal nodal metastasis, and propensity for stomal recurrence, as a result the prognosis of patients is always extremely unfavorable.^2^, ^25^ In this study, we confirmed the low 5-year OS and CSS rates of patients with subglottic SCC. Notably, patients with high-grade tumors or advanced-stage disease had significantly worse survival outcomes, suggesting that clinical stage and histologic grade are important factors predicting the long-term survival of patients with subglottic SCC. Interestingly, the prognosis of subglottic SCC remained similar over the past few decades, despite the progress in cancer treatment; however, it varied markedly over the past decades based on the treatment modality as reported, which possibly due to the differences in cohort sizes.^4^, ^11^, ^14^, ^25^^,^^31^

Patients with early-stage subglottic SCCs are often treated with a single treatment modality, whereas those at advanced-stage require a combination of multimodalities.^9^, ^16^, ^32^, ^33^ Starting with the landmark Veterans Affairs (VA) larynx trail, organ preservation protocols are increasingly being used for the treatment of laryngeal SCC.^34^ Thus, personalized and stratified treatment can occur in an organic and experimental fashion where patients can be shifted to appropriate regimens depending on their response to the therapy.^35^, ^36^ In light of its rarity, it has been difficult to draw conclusions on survival based upon treatment modality for subglottic SCC. Although non-surgical treatments were performed much frequently, we confirmed that surgery-oriented therapies, especially for the combination of surgery and radiotherapy, provided much favorable survival outcomes which lend credence to the conclusions proffered by Shi et al. as well.^27^ Few researches have focused on the application of chemotherapy on subglottic SCC regardless of its promising effects. We preliminary analyzed the survival outcomes of subglottic SCC treated with chemotherapy: the single use of chemotherapy or surgery plus chemotherapy failed to provide better survival outcomes which may attribute to insufficient distribution of cases in these regimens; while chemotherapy revealed positive effects to patients’ overall survival when combined with surgery or radiotherapy. CCRT has been verified to be of great help for preservation of larynx function, but we were unable to analyze its effect due to lack of specific data of chemotherapy and radiotherapy in SEER.^37^ Together, these findings demonstrate that the management of subglottic SCC can be highly variable and further studies would shed a light on the usefulness of multiple treatment regimens for subglottic SCC.

Currently, most patients with locally advanced subglottic SCC undergo a total laryngectomy, usually combined with neck dissection and, if necessary, thyroidectomy. Larynx preservation surgery is preferred for patients with early-stage subglottic SCC. Shaha et al. first reported long-term survival of early-stage subglottic SCC patients treated with organ preservation surgery, which was confirmed by another recent study as preservative laryngectomy provided up to 80% of 5-year disease-free for early-stage subglottic SCC.^7^, ^23^ Nevertheless, patients with advanced stage disease didn’t do well with organ-preservation therapies.^34^ In current study, patients who underwent total laryngectomy had the worst survival outcomes; by contrast, larynx preservation surgeries provided much favorable results. Further analysis between total laryngectomy and local tumor excision confirmed our findings after trimmed off partial laryngectomy subgroup (with only 25-cases) and adjusted for age and T staging that significantly influence the selection of surgeries for primary tumor, as well. These findings could be attributed to the fact that much patients treated with total laryngectomy had advanced-stage disease, yet larynx preservation surgeries were most commonly performed on patients at early-stage. The exacerbation of subglottic SCCs, for example advanced-stage and high-grade tumors, would eventually lead to less beneficial results from organ-preservation surgeries. It is still lack of further validation as to which type of surgery is much beneficial for the long-term prognosis since not only disease condition but also survival quality should be considered when caring patients with subglottic SCC. Of note, it was not until the middle 1990s that the efficacy of adjuvant therapy in advanced-stage laryngeal carcinoma was initially identified and accepted.^38^, ^39^ In this study, patients trended towards improved survival when received both surgery and adjuvant therapy, though without statistically differences. Conversely, Santoro et al. and Garas et al. uncovered a superior effect of surgery with adjuvant therapy in their single-institution reviews of subglottic SCC, respectively.^3^, ^9^ These discrepancies may have resulted from the distribution of treatment modality within the cohort, current findings may add to accumulating evidence that subglottic SCC patients would benefit from multimodality regimens, especially for advanced-stage cases. Taken together, our findings appear to corroborate previous discoveries with respect to the role for multimodality management of subglottic SCC with surgery and adjuvant therapy.

This study has several limitations: possible data errors and sampling errors may have been introduced due to the incomplete clinicopathological information and differences of case distribution on part of the patients. Miscoding in SEER may obfuscate details of cases. Additionally, the development of subglottic SCC’s management in recent years may affect our results. Finally, as the SEER database is an institution-based registry but not a true survey of the US population, the generalizability of our results should be tempered; further prospective multicenter randomized research in managing this disease is needed.

## Conclusions

In this retrospective, large cohort study, we confirmed the rarity of subglottic SCC, which was most common among males aged 60–70 years. Most patients were diagnosed with advanced-stage and low-grade disease. The prognosis of subglottic SCC remained poor in recent twenty-five years, despite the development in cancer therapies. The combination of surgery and adjuvant therapy improved the survival. Although patients with early-stage disease benefited from larynx preservation surgery, total laryngectomy provided favorable outcomes in patients with advanced lesions.

## Conflicts of interest

The authors declare no conflicts of interest.

## References

[1] Ferlito A., Rinaldo A. (2000). The pathology and management of subglottic cancer. Eur Arch Otorhinolaryngol..

[2] Dahm J.D., Sessions D.G., Paniello R.C., Harvey J. (1998). Primary subglottic cancer. Laryngoscope..

[3] Garas J., McGuirt W.F. (2006). Squamous cell carcinoma of the subglottis. Am J Otolaryngol..

[4] Strome S.E., Robey T.C., Devaney K.O., Krause C.J., Hogikyan N.D. (1999). Subglottic carcinoma: review of a series and characterization of its patterns of spread. Ear Nose Throat J..

[5] Jumaily M., Gallogly J.A., Gropler M.C., Faraji F., Ward G.M. (2021). Does subglottic squamous cell carcinoma warrant a different strategy than other laryngeal subsites?. Laryngoscope..

[6] Ampil F., Nathan C.A., Lian T., Baluna R., Milligan E., Caldito G. (2014). Postoperative management in laryngeal cancer with subglottic extension and histologically negative nodes: which patients need adjuvant radiotherapy?. Ear Nose Throat J..

[7] Shaha A.R., Shah J.P. (1982). Carcinoma of the subglottic larynx. Am J Surg..

[8] Levy A., Blanchard P., Temam S., Maison M.-M., Janot F., Mirghani H. (2014). Squamous cell carcinoma of the larynx with subglottic extension: is larynx preservation possible?. Strahlenther Onkol..

[9] Santoro R., Turelli M., Polli G. (2000). Primary carcinoma of the subglottic larynx. Eur Arch Otorhinolaryngol..

[10] Guedea F., Parsons J.T., Mendenhall W.M., Million R.R., Stringer S.P., Cassisi N.J. (1991). Primary subglottic cancer: results of radical radiation therapy. Int J Radiat Oncol Biol Phys..

[11] Paisley S., Warde P.R., O’Sullivan B., Waldron J., Gullane P.J., Payne D. (2002). Results of radiotherapy for primary subglottic squamous cell carcinoma. Int J Radiat Oncol Biol Phys..

[12] Spector M.E., Rosko A.J., Swiecicki P.L., Chad Brenner J., Birkeland A.C. (2018). From VA Larynx to the future of chemoselection: defining the role of induction chemotherapy in larynx cancer. Oral Oncol..

[13] Janoray G., Pointreau Y., Garaud P., Chapet S., Alfonsi M., Sire C. (2015). Long-term results of a multicenter randomized phase III trial of induction chemotherapy with cisplatin, 5-fluorouracil, ± docetaxel for larynx preservation. J Natl Cancer Inst..

[14] Hata M., Taguchi T., Koike I., Nishimura G., Takahashi M., Komatsu M. (2013). Efficacy and toxicity of (chemo)radiotherapy for primary subglottic cancer. Strahlenther Onkol..

[15] Landry D., Glastonbury C.M. (2015). Squamous cell carcinoma of the upper aerodigestive tract: a review. Radiol Clin North Am..

[16] Smee R.I., Williams J.R., Bridger G.P. (2008). The management dilemmas of invasive subglottic carcinoma. Clin Oncol (R Coll Radiol)..

[17] Porter G.C., Jayaraj S.M., Frosh A.C., Patel K.S. (1999). Submucosal squamous cell carcinoma of the subglottis. Otolaryngol Head Neck Surg..

[18] Mor N., Blitzer A. (2015). Functional anatomy and oncologic barriers of the larynx. Otolaryngol Clin North Am..

[19] Liu Y.H., Xu S.C., Tu L.L., Zhang K.L., Lu D.H., Zhang M. (2006). A rich lymphatic network exists in the inferior surface of the vocal cord. Surg Radiol Anat..

[20] Medina J.E., Ferlito A., Robbins K.T., Silver C.E., Rodrigo J.P., de Bree R. (2011). Central compartment dissection in laryngeal cancer. Head Neck..

[21] Chen A.Y., Halpern M. (2007). Factors predictive of survival in advanced laryngeal cancer. Arch Otolaryngol Head Neck Surg..

[22] Li H., Li E.Y., Kejner A.E. (2019). Treatment modality and outcomes in larynx cancer patients: a sex-based evaluation. Head Neck..

[23] Yu H., Tao L., Zhou L., Zhang M., Wu H., Li X. (2019). Results of surgical treatment alone for primary subglottic carcinoma. Acta Otolaryngol..

[24] Edge S.B., Byrd D.R., Brookland R.K., Washington M.K., Gershenwald J.E., Compton C.C. (2017).

[25] Marchiano E., Patel D.M., Patel T.D., Patel A.A., Xue Y.E., Eloy J.A. (2016). Subglottic squamous cell carcinoma: a population-based study of 889 cases. Otolaryngol Head Neck Surg..

[26] Gerry D., Fritsch V.A., Lentsch E.J. (2014). Spindle cell carcinoma of the upper aerodigestive tract: an analysis of 341 cases with comparison to conventional squamous cell carcinoma. Ann Otol Rhinol Laryngol..

[27] Shi L.L., McMullen C., Vorwald K., Nichols A.C., MacNeil S.D., Wadsworth J.T. (2021). Survival outcomes of patients with subglottic squamous cell carcinoma: a study of the National Cancer Database. Eur Arch Otorhinolaryngol..

[28] Lucioni M., D’Ascanio L., De Nardi E., Lionello M., Bertolin A., Rizzotto G. (2018). Management of paratracheal lymph nodes in laryngeal cancer with subglottic involvement. Head Neck..

[29] Timon C.V., Toner M., Conlon B.J. (2003). Paratracheal lymph node involvement in advanced cancer of the larynx, hypopharynx, and cervical esophagus. Laryngoscope..

[30] Kurita S., Hirano M., Matsuoka H., Tateishi M., Sato K. (1985). A histopathological study of carcinoma of the larynx. Auris Nasus Larynx..

[31] Coskun H., Mendenhall W.M., Rinaldo A., Rodrigo J.P., Suárez C., Strojan P. (2019). Prognosis of subglottic carcinoma: Is it really worse?. Head Neck..

[32] Chiesa F., Tradati N., Calabrese L., Zurrida S., DePaoli F., Costa L. (2001). Surgical treatment of laryngeal carcinoma with subglottis involvement. Oncol Rep..

[33] MacNeil S.D., Patel K., Liu K., Shariff S., Yoo J., Nichols A. (2018). Survival of patients with subglottic squamous cell carcinoma. Curr Oncol..

[34] Department of Veterans Affairs Laryngeal Cancer Study Group, Wolf G.T., Fisher S.G., Hong W.K., Hillman R., Spaulding M. (1991). Induction chemotherapy plus radiation compared with surgery plus radiation in patients with advanced laryngeal cancer. N Engl J Med..

[35] Urba S., Wolf G., Eisbruch A., Worden F., Lee J., Bradford C. (2006). Single-cycle induction chemotherapy selects patients with advanced laryngeal cancer for combined chemoradiation: a new treatment paradigm. J Clin Oncol..

[36] American Society of Clinical Oncology, Pfister D.G., Laurie S.A., Nathan C.A., Adelstein D.J., Eisbruch A. (2006). American Society of Clinical Oncology clinical practice guideline for the use of larynx-preservation strategies in the treatment of laryngeal cancer. J Clin Oncol..

[37] Forastiere A.A., Goepfert H., Maor M., Pajak T.F., Weber R., Morrison W. (2003). Concurrent chemotherapy and radiotherapy for organ preservation in advanced laryngeal cancer. N Engl J Med..

[38] Vermund H. (1970). Role of radiotherapy in cancer of the larynx as related to the TNM system of staging. A review. Cancer..

[39] Bonomi M.R., Blakaj A., Blakaj D. (2018). Organ preservation for advanced larynx cancer: A review of chemotherapy and radiation combination strategies. Oral Oncol..

